# Edible oleogels for oral delivery of berberine in dairy food: In‐vitro digestion study

**DOI:** 10.1002/fsn3.3994

**Published:** 2024-04-05

**Authors:** Mozhdeh Sarraf, Adel Beigbabaei, Sara Naji‐Tabasi

**Affiliations:** ^1^ Department of Food Chemistry Research Institute of Food Science and Technology (RIFST) Mashhad Iran; ^2^ Department of Food Nanotechnology Research Institute of Food Science and Technology (RIFST) Mashhad Iran

**Keywords:** berberine, fat replacer, *Ocimum bacilicum* L., whey protein concentrate, xanthan gum

## Abstract

Oleogel is a viscoelastic, spreadable and semi‐solid structure, which is used as a fat substitute and a controller the release of bioactive compounds. The aim of this study was to develop low fat dairy dessert enriched with berberine with applying oleogel system as delivery system and fat replacer. The oleogel prepared with an emulsion‐templated methods based on soluble interaction of whey protein concentrate (WPC), WPC‐basil seed gum (BSG), and WPC‐xanthan gum (XG). In the first step, berberine release kinetic in in‐vitro gastrointestinal environment was studied. The results showed that the mouth environment had the highest release rate of berberine. Cooperation of hydrocolloids in oleogel increase stability of structure in stomach condition in compared with WPC oleogel. The suitable model to fit the oleogels contain beberine was the Korsmeyer‐Papas that was the highest *R* ^2^ (.98). According to release results of berberine from oleogel network, the oleogel 0.6BSG:WPC was chosen and applied in formulation of dairy dessert at different levels (0%, 25%, 50%, 75% and 100% of oleogel) instead of cream. The dessert contained uncoated berberine had the unacceptable bitterness in comparison with samples containing coated berberine with oleogel. The overall acceptance decreased with increment of oleogel due to increasing of bitter taste. Appling berberine (therapeutic compound) and oleogel (fat‐substitute) to achieve marketable consumer products showed positive effects on trend of the study, especially at low level of substitution.

## Graphical abstract

The sample containing 0.6BSG:WPC has controlled release of berberine in in‐vitro gastrointestinal environment and can be used as a fat replacer in dairy food. Using berberine (therapeutic compound) and oleogel (fat‐substitute) to achieve marketable consumer products showed positive effects.

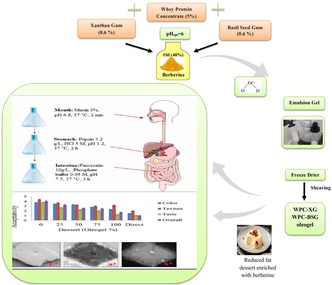

## 1 INTRODUCTION

Berberine is a quaternary ammonium protoberberine alkaloid with an isoquinoline scaffold which was isolated from *Xanthoxylon cava in* 1826 (Patel, 2021). Berberine has widely distributed in several medicinal herbs and found in the barks, roots, and stems of plants, such as *Coptis chinensis*, *Phellodendron amurense*, *Hydrastis canadensis*, *Berberis* types and *Coccinia fenestratum* (Jin et al., 2016; Neag et al., 2018; Patel, 2021). The alkaloid has broad spectrum of pharmacological activities. So it has been used in traditional medicine since ancient times. A lot of research has been done on the therapeutic properties of berberine that indicated it has effects on cancer, obesity, diabetes, inflammation, bacteria problem, hepatoprotective activity atherosclerosis, diarrhea, depression Alzheimer's disease, rheumatoid arthritis, neurodegenerative diseases and cardiovascular diseases (Caliceti et al., 2016; Chang et al., 2016; Chang et al., 2015; Jiang et al., 2015; Joshi et al., 2011; Kumar et al., 2015; Peng et al., 2015).

Yellowish crystalline powder, odorless with an extremely bitter taste is the physical characteristics of berberine. It is relatively soluble in methanol, slightly soluble in ethanol, and very slightly soluble in water (Dostál et al., 2004).

Although no side effects have been observed with overdose, there is not enough information to show that long‐term use is safe. Berberine can be caused upset stomach, constipation, nausea, headache, low blood pressure in some people. Also it is harmful on fetus and pregnant women (Mehrabani et al., 2019) have to avoid taking berberine orally. Therefore, the dosage of this compound is very important in industries (Chen et al., 2020). The safety dosage of berberine in oral administration in mice is 20.8 g/kg and in human would be 2.97 g/kg which is 100 times above the typical prescribed doses in clinical trials studies (Kheir et al., 2010).

Despite having valuable therapeutic properties of berberine, there is a limitation use in various industries due to its bitter taste. According to the studies done in the past years, the most common use of this alkaloid has been in the pharmaceutical industry, such as the production of creams, or capsules. On the other hand, it has been paid less attention in the food industry. Therefore, in order to use this valuable compound in the food industry, one of the ways to remove the bitter taste of berberine is to use delivery systems. There are various methods for delivery of berberine, one of them is the oleogel system. Oleogels are self‐standing systems that are able trap edible liquid oil into a tridimensional network. They also help to use less of fat by formation the crystallization oleogelators (Patel et al., 2015) without changing the chemical composition (Öğütcü & Yılmaz, 2015), increasing firmness, encapsulation and control release of bioactive components (Li et al., 2021; O'Sullivan et al., 2016). A common used method to produce oleogel is the emulsion‐templated method (Romoscanu & Mezzenga, 2006). To prepare an oil‐in‐water emulsion can be applied proteins as an emulsifier and polysaccharides are used to reinforce the interface. Sufficient stability of an interface like polysaccharides is important in the step of water removing that causes a high internal phase emulsion (HIPE) including a three‐dimensional network of polymers filled with oil to form. Then the water of emulsions are removed by drying in an oven, freeze‐drying, or at ambient temperature method (Abdolmaleki et al., 2020; Patel et al., 2015; Qiu et al., 2018; Romoscanu & Mezzenga, 2006; Tavernier et al., 2017; Wijaya et al., 2019).

On the other hand, oleogels are one of the most popular systems for production low fat food. Due to the increased attention to diets and compounds to replace solid fat, oleogels have the potential to be used in products such as chocolate, confectionery, dairy desserts and ice cream. The results obtained in the industry in the last decade show the success of using this combination and the commercial potential of the oleogel system (Hughes et al., 2009). Food and Agriculture Organization (FAO) encourages industries to decrease the consumption of saturated fatty acids due to health‐related concerns (FAO, 2010).

Even though these research show that proteins on their own can be sufficient to generate a stable system, the mixture of proteins and polysaccharides causes stability to increase more. In these systems, the oil itself is not structured, but is held together in a network of proteins and polysaccharides (Feichtinger & Scholten, 2020). In this study, were used xanthan gum (XG) which is created by *Xanthomonas campestris*, and basil seed gum (BSG) which is extracted from the Basil seeds (Sarraf et al., 2023), and whey protein concentrate (WPC) to prepare oleogels. Considering that berberine dissolves easily in oil, this structure was used to cover the bitterness of berberine.

Overall, this study was conducted with the aim of applying a valuable compound, the native biopolymers and preparing a diet production in the food industry. Therefore, two processes were followed. The rate of berberine release was evaluated from the choice oleogel systems in the simulated environment of the gastrointestinal tract. On the other hand, the effectiveness of oleogel was investigated in order to remove the bitterness of berberine and also as a fat substitute in popular products such as dairy desserts.

## 2 MATERIALS AND METHODS

### 2.1 Materials

Basil seeds were purchased from a traditional market and the gum (BSG) was prepared based on Razavi and Naji‐Tabasi (2017). Xanthan gum (XG) and Whey protein concentrated (WPC) contained 70% proteins, berberine standard (97%) were prepared from Sigma‐Aldreich, (Germany), Westland (New Zealand), and Xi'an Saiyang Bio technology Co., (China), respectively. HCl, NaOH, and CaCl_2_ were supplied from Merck Co. (Germany). Commercial sunflower oil was used for the preparation of the olegels. Enzymes were prepared from Merck Co. (Germany) and also dialysis bag was purchased from MEMBRA‐CEL Co. (Germany). Deionizer water purified was used for the preparation of the solution.

### 2.2 Methods

#### 2.2.1 Preparation of oleogel by emulsion‐templated method

After dispersion of the biopolymers in deionized water (WPC: 5% w/w and hydrocolloids: 0.6% w/w), they were left overnight for hydration completely. For preparation of emulsion gels, suspensions of XG:WPC (1:1) and BSG:WPC (1:1) were prepared and then pH of mixture was set on 6 by HCl and NaOH (pH meter Metrohm, Switzerland). The solutions were placed in a heating circulator (Julabo, EH 19, USA) at 85°C for 30 min and cooled immediately.

To prepare emulsion gel, 40% sunflower oil (Iran) and 0.06% w/w berberine powder (based on oleogel weight) was slowly added to the biopolymer complex and pre‐homogenized with an ultraturrax (IKA, TG25, Germany) at 10,000 rpm for 120 s and then homogenized (HL1.2, HST, Germany) under pressure 20 MPa for 150 s. After reaching to room temperature, 10 mM CaCl_2_ was added to emulsion. Then, the emulsion gel was transferred to flasks of freeze drier (FDU‐8606, OPERON, South Korea) and was kept at −50 ± 5°C under vacuum 10^−4^ Torr for 72 h (Patel et al., 2015). The end of step, the sample was sheared (RZR 2102 control, Heidoph, Germany) for 30 s to obtain desired structure of oleogel.

#### 2.2.2 In vitro berberine release studies

Berberine release from the oleogel structure was examined in the mouth after 120 s in pH 6.8, stomach at pH 1.2 after 2 h, and intestine at pH 6.8 after 3 h. The process was performed on a shaker incubator (100 rpm).

The simulated mouth environment contained 3% mucin in pH 6.8, the stomach contained 3.2 g/L pepsin with 5 M hydrochloric acid in pH 1.2, and the intestine contained 10 g/L pancreatin and 0.05 M phosphate buffer in pH 7.5 (Solghi et al., 2020; O'Sullivan et al., 2016). Dialysis bags (10–12 kDa) were used to evaluate the delivery of berberine. The bags were placed in deionized water at room temperature for overnight to completely remove any preservatives, before using them. 3 g of oleogel was placed in the bags and immersed in oral medium.

Berberine release from the oleogel structure was examined in the mouth after 120 s. Then the bag was transferred to the simulated gastric environment. For simulated stomach (for 2 h) and intestinal (for 3 h) environments, 4 mL was sampled every 30 min and an equal volume of buffer solution was added to the medium. The process was performed on a shaker incubator (100 rpm) at temperature 37°C. The absorbance of each sample was measured by a spectrophotometer (Hach, Germany) at 348 nm and evaluated by a standard curve (*R* ^2^ = .996) (Sabouri et al., 2017).

#### 2.2.3 Berberine release kinetic

The kinetic of the data obtained from the release of berberine in vitro were evaluated using several standard models such as zero (Equation 1), first (Equation 2), second order (Equation 3), Higuchi (Equation 4), and Korsmeyer‐Papas models (Equation 4) (Ghitman et al., 2018).

(1) $$Q_{0}-Q_{t}=k.t\;\text{Zero order model}$$

(2) $$Q=Q_{0}.e^{\mathrm{kt}}\;\text{First order model}$$

(3) $$1/Q_{t}=k.t+1/Q_{0}\;\text{Second order model}$$

(4) $$Q=k.t^{0.5}\;\text{Higuchi model}$$

(5) $$Q=k.t^{n}\;\text{Korsmeyer}-\text{Peppas model}$$

where *Q* is the amount of berberine released at time *t*, *Q*_0_ is the initial amount of *Q*, *k* is the rate constant, *n* is diffusional exponent (indicating the berberine release mechanism), *a* is the time constant, and *b* is the shape parameter.

#### 2.2.4 Preparation of dairy dessert

Five formulations were prepared with different percentages of oleogel (0%, 25%, 50%, 75% and 100% w/w) substituent of the cream. The amounts of 82.1% (w/w) milk (3% fat) and 4.8% (w/w) cream (30% fat), and 11.49% (w/w) of sugar were mixed and placed in a water bath at 40°C for 10 min to hydrate the ingredients. During this time, the mixture was continuously stirred. Then the samples were stayed in the bath at 90°C for 10 min. After that, the samples were placed in an ice bath to cool 40°C. Then, vanilla (0.11% w/w) and rose water (1.5% w/w) were added and stirred for 1 min. Finally, the samples were stored at 4°C (Seuvre et al., 2008).

#### 2.2.5 Physicochemical analysis of dairy dessert

The physicochemical and mechanical of desserts containing 0 (D0), 25 (D25), 50 (D50), 75 (D75) and 100% (D100) oleogel were investigated.

For evaluation of the amount of desserts syneresis, Ghiyasi et al., 2016 method was used (Ghiyasi et al., 2016).

Determination of berberine content was done by High‐performance liquid chromatography (HPLC), Shimadzo LC‐ 2010 HPLC system (Kyoto, Japan), with C‐18 (5.9 × 1.81 inch) column and particle size 10 nm. the method was performed.

The textural properties of dairy desserts were studied using a texture analyzer (Stable Micro System, TA‐XT Plus) by TPA method at 25°C. In this test, a stainless steel probe P/75 (England) with a diameter of 80 mm with rate of 1 mm.s^−1^, Trigger force 3 g and 70% strain was used. The parameters of hardness, consistency, adhesiveness, and apparent elastic modulus were evaluated (Ghiyasi et al., 2016).

The colors of desserts were measured using computer version system with five fluorescent lights (Opple, 8 W, model: MX396‐Y82; 60 cm in length) with a color index (Ra) close to 95%. The system comprised of a digital camera (Canon EOS 1000D, Taiwan) with lens focal length of 35 mm for color analysis and 45 mm, image capturing box a wooden box, 45 cm above the sample and at the angle of 458 with sample plane to give a uniform light. Version (6.1.1) was used to acquire the images in the computer in TIFF format. The sample was placed into a plate. Parameters L*, a* and b* of prepared image (resolution of 300 dpi) with bmp format was evaluated by the Image J software.

#### 2.2.6 Organoleptic properties of dairy dessert

The organoleptic characteristics of the desserts were evaluated with the 5‐point hedonic method (parameter include taste, texture, color, and overall). In the organoleptic evaluation, all desserts were compared with a dessert containing uncoated berberine (Direct, 0% oleogel‐full fat), without oleogel (D0‐witness).

#### 2.2.7 Statistical analysis

The complete randomized design was used and the analysis of data was done by SPSS version 11.0 using one‐way analyses of variance (ANOVA). Three replications were conducted for each test and the significant differences were analyzed with Duncan's multiple range test. The differences are considered to be statistically significant when the *p* values are <.05. The curves plotted by the software Excel 2007 and Origin Lab 2017.

## 3 RESULTS AND DISCUSSION

### 3.1 In vitro release of berberine from the oleogel systems

Digestion is the breakdown of large food particles into smaller absorbable nutrients needed for energy production, growth and cell repair.

**FIGURE 1:**
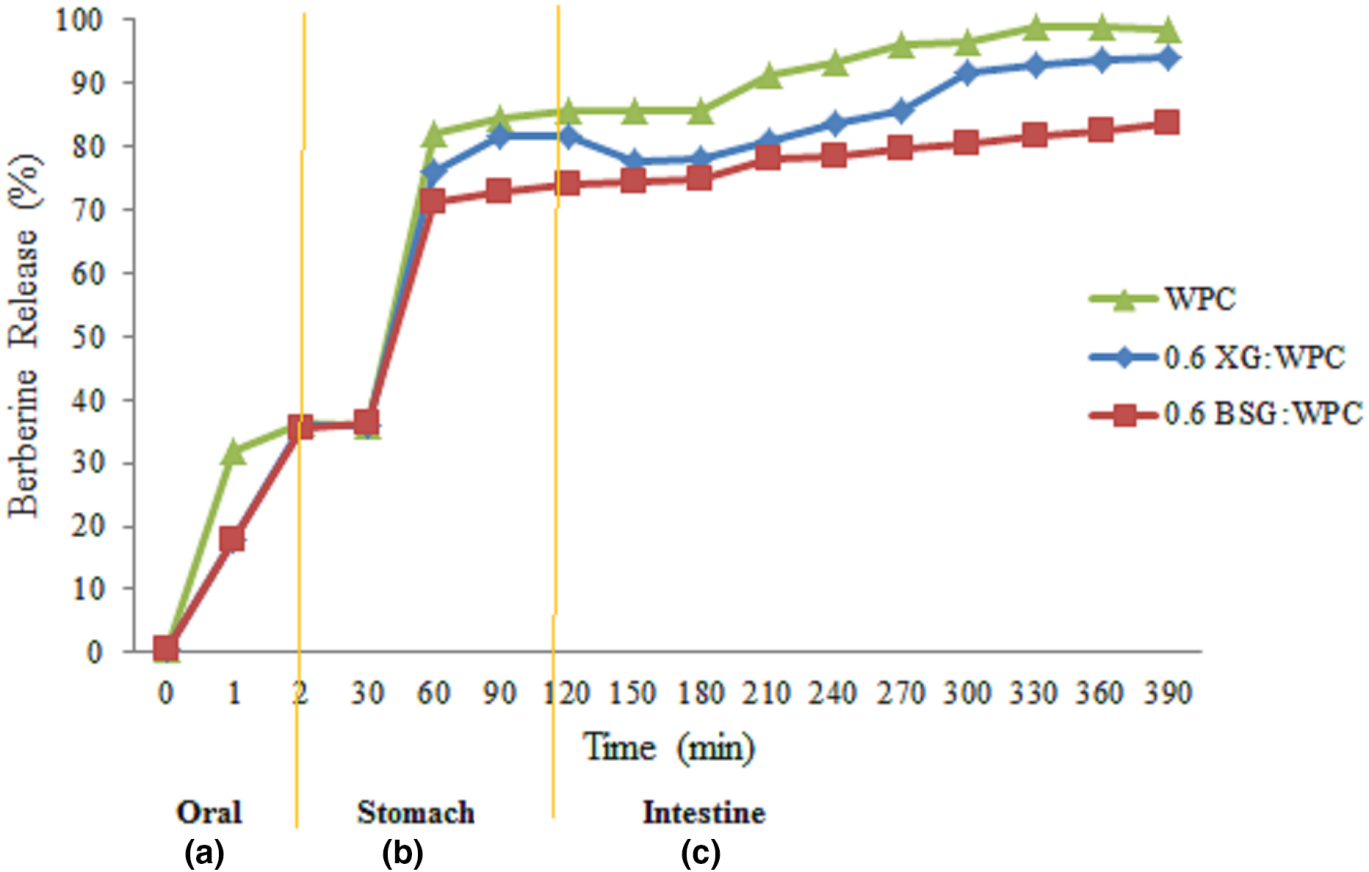
Berberine release from oleogels in (a) oral (b) stomach (c) intestine environment.

The berberine release from the oleogel system was investigated in three simulated environments of the mouth, stomach and intestine was shown in Figure 1.

According to Figure 1, a rapid release of berberine took place in the mouth environment and about 30% of berberine was released from oleogel system. There are several variables in retaining or releasing bioactive compounds from the oleogel system in the mouth phase, including mouth temperature, saliva composition and flow rate, pH and ionic strength, frictional forces, mixing conditions, duration and enzyme activity (de Roos, 2003; Salles et al., 2007). The release rate of berberine from the WPC oleogel in mouth environment was 36.11%, while in the samples containing 0.6XG:WPC and 0.6BSG:WPC was 35.70% and 35.55%, respectively.

After that, the treatments were transferred to the gastric environment. As can be seen in the Figure 1, the highest amount of release occurred in all the samples in the first 30 min and then it continued with a gentle slope. Also, a higher release percentage took place in the WPC oleogel compared to the other two samples, so that at the end of 2 h, the release content of berberine in WPC, 0.6XG:WPC and 0.6BSG:WPC oleogels was estimated about 85.55, 81.50, and 73.88, respectively. The release rate of berberine from the oleogel structure in all samples decreased after 30 min and the release occurred at a lower rate. The stomach plays an important role in the initial steps of food digestion. In this phase, gastric acid, whose main components include water, hydrochloric acid, pepsin and basic compounds, is secreted. Pepsin is the main factor in protein digestion and breaks them down into smaller peptides and amino acids. Hydrochloric acid is another component of gastric acid and plays an important role in creating the pH required for pepsin activity. A liquid mixture of partially digested food particles enters the intestine through the action of pepsin and the property of squeezing the stomach (Heda et al., 2019).

The lower strength of the oleogel containing WPC compared to the other oleogels, indicates that polysaccharides played an effective role on the increasing strength of oleogel structures and controlling the release of berberine. According to the results of texture analysis on their primary emulsion gels and oleogles, hardness, consistency of the structure were raised of in line with increasing the concentration of polysaccharides, this event was not far from expected (Sarraf et al., 2023). It also seems that the amount of acid strength is also very effective on berberine release. The zeta potential of WPC emulsion gel is −22.40 mV according to previous study and changes to the more negative value with the increase of anionic polysaccharides on the surface of the particles, so that the zeta potential of 0.6BSG:WPC and 0.6 XG:WPC emulsion gels are −28.50 and −26 mV, respectively (Sarraf et al., 2023).

Therefore, when WPC oleogel is placed in the stomach environment, which has a pH of about 1.2, it will cause repulsion between the structures and will result in the release of berberine. While the presence of polysaccharide causes the structure to be close to each other and increases the strength of the network in this environment and the exit of berberine occur slowly.

In the simulated environment of the intestine, the highest rate of diffusion occurred in the first minutes. In general, the release of berberine occurred in all oleogels with different rates and amounts, and at the end of the intestine, the release rate was fixed, and it was determined that the highest release rate was in the sample WPC, and the effect of polysaccharides, especially BSG gum, on creating a strong structure for oleogels was proven. The lowest release rate was related to the 0.6BSG:WPC oleogel (83.69%). This amount in WPC and 0.6XG:WPC was estimated 98.50% and 94.20%, respectively.

Naji‐Tabasi et al. (2017) reported that the encapsulation of bioactive materials inside anionic gels significantly increases the efficiency and targeted binding of core materials. Also, nanoparticles are swollen and contracted according to the pH and ionic strength of the environment and are effective factors in the targeted release of substances enclosed in nanoparticles (Naji‐Tabasi et al., 2017). Chloe et al. (2017) investigated the effect of oleogelation on the bioavailability of beta‐carotene and the results showed that gel structure declined leakage oil and vitamin into the simulated environment. Abdollahi (2018) reported that the use of oleogel had a positive effect in comparison with oil. Also, the molecular weight of the oleogelators used affects the release rate (Abdollahi, 2018; Chloe et al., 2017).

Considering that the main compound of the oleogel structure was based on protein, the release of berberine was under the influence of the protein, and in intestinal phase, the release from WPC oleogel was more than 0.6XG:WPC and 0.6BSG:WPC. Therefore, the more controlled release of berberine coated by oleogel containing 0.6BSG:WPC compared to other treatments was an expected result. According to the obtained results, 0.6BSG:WPC oleogel was chosen for the preparation of dairy dessert.

### 3.2 Synthetic of berberine release from the oleogel system

The release kinetics of berberine from the oleogel system was investigated by different models. It means that the data obtained from the release of berberine in the mouth, stomach, and intestine environment were fitted based on the models mentioned in section 2–4 and can follow several synthetic mechanisms. According to the data fitting comparison, it can be concluded that berberine release follows the Korsmeyer‐Peppas method that was the highest *R* ^2^ (.98).

According to Table 1, the results showed that the release rate (*k*) of berberine in the stomach was higher than the intestine in all oleogels. On the other hand, it was observed that the lowest release rate was related to oleogel 0.6BSG:WPC, which indicates the greater resistance of this gum against low pH of the stomach environment.

**TABLE 1:** Release kinetics of berberine from oleogel system.

Model	Parameter	WPC		0.6 BSG:WPC		0.6XG:WPC	
		Gastric phase	Intestine phase	Gastric phase	Intestine phase	Gastric phase	Intestine phase
Korsmeyer‐Peppas	*k*	6.5	0.02	5.48	0.07	5.54	0.11
	*n*	0.2	0.95	0.05	0.57	0.13	0.64
	*R* ^2^	.99	.98	.98	.99	.99	.99
	RMSE	0.45	0.21	0.45	0.06	0.33	0.05

Abbreviations: BSG, basil seed gum; WPC, whey protein concentrate; XG, xanthan gum.

In addition, by considering the diffusion power parameter (*n*), expressing the type of berberine diffusion mechanism, it can be seen that the release was made through one type of mechanism or a combination of several processes. If the transport exponent (*n*) is less than 0.45, the release mechanism is of the Fick diffusion type and indicates that the size of the particles is smaller than the holes in the wall of the oleogel. Release is two‐phase transition, which is due to the swelling of the oleogel system. Parameter (n) of Fick's release is 0.45 (Siepmann & Peppas, 2012). According to Table 1, the value of (*n*) in the stomach environment 0.04, 0.05 and 0.07 for WPC, 0.6BSG:WPC and 0.6XG:WPC, respectively. It is indicated that Fick model for stomach, while it was the non‐Fick in intestine system, which is actually the diffusion mechanism along with erosion and the removal of the wall. It can be showed that the release of berberine has been out of control of one process. Also, by studying transport constant (k coefficient), it can be seen that the rate of diffusion in the stomach is much higher than in the intestine. Protease, high‐strength salt ions and changing pH in small intestine digestive juice may gradually induce disintegration of protein‐polysaccharide colloidal particles, which facilitates displacement by bile salts and adsorption of lipase‐colipase (Sun et al., 2022).

There is a possibility that the oleogel structure is unstable due to the presence of high protein in the structure compared to the pH used in the stomach, which is about 1.2, and has caused the structure to disintegrate and a percentage of berberine to leave the structure.

### 3.3 Physicochemical properties of dairy dessert

Table 2 shows the content of berberine of each dairy dessert. The berberine content increased with increasing the percentage of oleogel and the most content was related to dessert 100% (10.61 mg/L).

**TABLE 2:** Synersis, fat content and color parameters of dairy desserts with different percentages of oleogel (0%, 25%, 50%, 75% and 100%) containing berberine.

Dessert (oleogel %)		Parameter				
		D0	D25	D50	D75	D100
Berberine (mg.L)		0	5.41 ± 0.02^d^	6.50 ± 0.20^c^	7.88 ± 0.02^b^	10.61 ± 0.80^a^
Synersis (%)		15.70 ± 0.05^a^	12.80 ± 0.02^b^	2.99 ± 0.02^c^	1.90 ± 0.03^e^	2.10 ± 0.03^d^
Fat (%)		24.10 ± 0.10^a^	23.50 ± 0.09^b^	19.6 ± 0.09^c^	14.60 ± 0.20^d^	12.07 ± 0.40^e^
Color	L*	3.04 ± 0.07^e^	5.70 ± 0.40^d^	7.91 ± 0.12^c^	8.97 ± 0.33^b^	11.51 ± 0.20^a^
	a*	−0.49 ± 0.03^a^	−0.96 ± 0.11^b^	−2.02 ± 0.04^c^	−2.63 ± 0.11^d^	−3.22 ± 0.12^e^
	b*	78.09 ± 0.13^a^	76.85 ± 1.11^b^	76.82 ± 0.16^b^	76.35 ± 0.55^c^	75.57 ± 0.25^d^

*Note*: Different letters indicate significant differences between samples in row at *p* < .05 by Duncan's multiple range test.

* Max amount letter: a.

The fat content of the dessert decreased as a result of the lower fat content in the oleogel when compared to that of dessert 0% (*p* < .05). The highest and lowest fat content were related to the dessert 0 (24.1%) and complete replacement of oleogel (12.07%), respectively, so that, fat content was reached to 50% in desert with replacing 100% oleogel (D100) compared to the full‐cream sample (D0) that unsaturated fat has replaced saturated fat, which is an important point (Table 2). This can also affect the syneresis of the product and increase the ability to hold water in the dessert structure by reducing the amount of oil. Therefore, this system can be used as a fat substitute in popular products such as desserts.

The presence of different percentages of oleogel in the dessert had a positive effect on gel network; and leaving free water from the structure was decreased after half an hour with increasing oleogel replacement content, so that, the syneresis of dessert D0 and the dessert D100 were 15.7% and 2.1%, respectively (Table 2). The amount of syneresis of the samples is related to the mechanical resistance of the protein network. Adding dry matter and hydrocolloids to formulation are a common method to prevent syneresis and low gel strength of dairy products (Brückner‐Gühmann et al., 2019; Sahan et al., 2008). Syneresis is a natural phenomenon that free excess water is separated from the gel network during a certain period of time. This process is undesirable in products such as dairy desserts, and as a result, the rearrangement of the gel network has occurred and caused an increase in the connections of particles, and therefore the network tends to wrinkle and the internal water exits to the outside (Omayma, & Youssef, 2007). Ghiyasi et al. (2016) reported that the parameter is related to the hardness, consistency of the texture, size and type and location of the network pores (Ghiyasi et al., 2016). Therefore, the oleogel in the dessert has created a structure with a more suitable consistency and has reduced the water release.

An increase in the oleogel levels, increased brightness and resulted in the production of lighter colors from L* analysis in the dessert structure. Factor a* also showed a downward trend with increasing percentage of oleogel containing berberine and the color shifted to red color, so that the highest value in the sample D0 (−0.49) and the lowest value for D100 (−3.22) was observed. The b* factor also increased as the oleogel level increased. This change in color spectrum from red to green can be related to the color of BSG, which has a dark color.

### 3.4 Textural properties of dairy dessert

Textural properties of the dessert were investigated and showed in Table 3. The hardness parameter reflected strength of the gel structure under compression (Sarraf et al., 2023). The results illustrated that the highest hardness is related to the D0 which can be related to the high content of cream in the structure, so that it causes a strong structure in comparison with desserts containing oleogels to generate. The hardness of free‐oleogel dessert (D0) was 974.81 g, whereas the hardness of dessert containing 100% oleogel (D100) was 735.41 g. The presence of soluble interaction of protein‐hydrocolloids oleogel has a positive effect on textural properties and can produce a variety of products by creating aqueous connections (Zhang et al., 2024).

**TABLE 3:** Textural properties of dairy desserts with different percentages of oleogel (0%, 25%, 50%, 75% and 100%) containing berberine.

Dessert (oleogel %)	Parameter				
	Hardness (g)	Adhesiveness (g.s)	Consistency (g.s)	Springiness (mm)	Apparent elasticity modulus (g.s^−1^)
D0	974.81 ± 5.20^a^	−247.76 ± 12.30^e^	4010.19 ± 19.20^a^	11.43 ± 0.19^a^	93.05 ± 9.70^a^
D25	771.50 ± 12.10^b^	−251.17 ± 10.00^d^	3978.42 ± 17.20^b^	11.40 ± 0.10^b^	75.82 ± 13.00^b^
D50	755.25 ± 8.80^d^	−270.04 ± 9.30^c^	3279.04 ± 32.10^c^	11.42 ± 0.80^a^	75.47 ± 10.10^c^
D75	758.38 ± 7.60^c^	−325.11 ± 10.70^b^	2877.45 ± 14.10^e^	11.12 ± 0.60^d^	74.75 ± 5.10^d^
D100	735.41 ± 8.90^e^	−372.73 ± 17.40^a^	2889.10 ± 5.40^d^	11.35 ± 0.20^c^	70.00 ± 3.90^e^

*Note*: Different letters indicate significant differences between samples in row at *p* < .05 by Duncan's multiple range test.

Apparent elasticity modulus is the ratio of stress to strain in the elastic range and it is in line with the hardness parameter (Razavi & Akbari, 2009; Shirvani et al., 2014).Thus, as seen in Table 3, apparent elasticity modulus decreased with increasing oleogels to formulation of dessert, so that the parameter in sample D0 was indicated 93.05 g.s^−1^, while it was 66.65 g.s^−1^ in D100. But the amounts were close to each other in sample 25%, 50% and 75% (~75 g.s^−1^).

The other important parameter is adhesiveness, which is a measure of the forces that keep the product adhered to the surface of another material and introduces the flow capability or liquid state of materials (Adhikari et al., 2001; Frabetti et al., 2021). The adhesion index was also statistically significant and a decreasing trend was observed. The D0 and D100 showed the lowest (−288.14 g.s) and highest (−361.79 g.s) adhesiveness, respectively. Naji‐Tabasi et al. (2020) reported that the oleogel containing more protein had more adhesiveness compared to other oleogels (Naji‐Tabasi et al., 2020), so it can be effective on texture properties of final production.

Consistency is the energy required to achieve a specific shape change and strength of the internal bonds of matter. The parameter indicated the gravitational forces between the particles to prevent the splashing of the structure (Poursani et al., 2021). The different percentage of oleogel in desserts effected on the cohesion properties and caused the parameter to increase, so that the sample replaced with 100% oleogel (consistency = 0.24) was significantly different from other treatments.

The cream in formulation helps more texture cohesion of the structure. But the trend of this parameter is forward with increasing amount of oleogel that is indicate effect of basil seed gum on interaction of primary complex.

### 3.5 Organoleptic evaluation of dairy dessert

**FIGURE 2:**
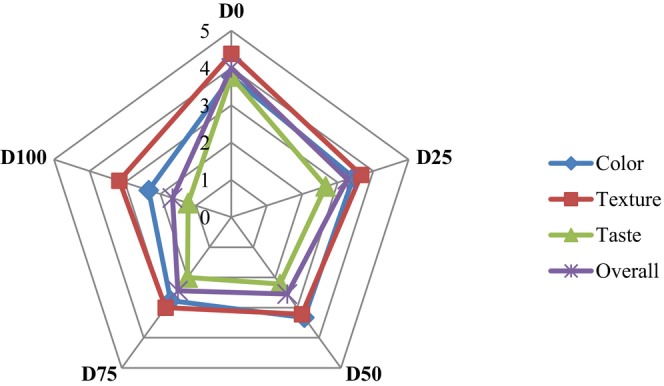
Organoleptic properties of reduced fat dairy desserts with different percentages of oleogel (0%, 25%, 50%, 75% and 100%) containing berberine, and uncoated berberine (direct).

As can be seen in Figure 2, shifting color from white to yellow is the result of reduction in desirability because of the addition of oleogel. The highest score was obtained by the D0. The highest levels of oleogel (D100) achieved a lower score which is due to bitterness created by berberine, which caused a bitter aftertaste. The fat content of the samples was also reduced by replacing the oleogel with cream, and the results showed that the tendency was towards samples with a higher fat content and creamy taste. Also, in the samples containing berberine without the oleogel (D0‐witness) system, the dairy dessert was not acceptable in the mouth, and consumers did not feel the necessary softness after consumption, in addition to the unacceptable bitterness in the product. Also, the white color of the desserts approached yellow with the increase of oleogel, and the high score of the sample D0 indicates the acceptability of creamy color for dairy desserts. Also, the amount of fat in the samples was reduced by substituting oleogel instead of cream, and the results showed that there is a tendency towards samples with a higher amount of cream. But the important point was related to the sample D0. Naji‐Tabasi et al. (2020) reported that decrease of the oil content causes the taste to intensify and also the aroma will be reduced (Naji‐Tabasi et al., 2020). The D0‐witness dessert, created a very noticeable bitterness in the mouth, which was unacceptable by the control panel. This indicates the positive effect of the oleogel structure in controlling the bitter taste of berberine, so that the oleogel structure has been able to cover the berberine granules relatively well and reduce the perception of berberine bitterness during consumption.

Figure 2 shows that the control sample obtained the highest score in terms of overall acceptability though a significant difference between this, followed by the dessert D25 (*p* < .05).

According to the obtained results, this system can be used as a fat replacement in popular products such as desserts.

## 4 CONCLUSION

This research was done with three main goals: Enriching popular products with valuable compounds such as berberine, using oleogel as a fat substitute and introducing plant‐based gums as a natural potential with wide resources in the world. According to the result of the release of berberine from oleogel system in the simulated environment of the digestive system, the highest release rate was observed in the mouth and the lowest release rate was related to BSG oleogel. 0.6BSG:WPC was used as a replacement in dairy dessert. D100 dessert (sample with the highest amount of oleogel) had the least acceptance. The hardness of D0 and D100 desserts were 974.81 and 736.41 g, respectively. Overall, using berberine (therapeutic compound) and oleogel (fat‐substitute) to achieve marketable consumer products showed positive effects on trend of the study. According to the results WPC‐basil seed gum oleogel can improve the textural properties of reduced fat food. As a result, it is possible to use this structure for development functional food with low fat. However, more investigations should be conducted to use it in the process and high‐scale production.

## AUTHOR CONTRIBUTIONS

**Mozhdeh Sarraf:** Investigation (equal); software (equal); writing – original draft (equal). **Adel Beigbabaei:** Supervision (equal); validation (equal); writing – review and editing (equal). **Sara Naji‐Tabasi:** Conceptualization (equal); project administration (equal); supervision (equal); validation (equal); writing – review and editing (equal).

## CONFLICT OF INTEREST STATEMENT

The authors declare that they have no competing interests.

## ETHICS APPROVAL

Not applicable.

## Data Availability

Data available on request from the authors.
